# Soluble Neuregulin1 Down-Regulates Myelination Genes in Schwann Cells

**DOI:** 10.3389/fnmol.2018.00157

**Published:** 2018-05-14

**Authors:** Marwa El Soury, Benedetta E. Fornasari, Michela Morano, Elio Grazio, Giulia Ronchi, Danny Incarnato, Mario Giacobini, Stefano Geuna, Paolo Provero, Giovanna Gambarotta

**Affiliations:** ^1^Department of Clinical and Biological Sciences, University of Torino, Turin, Italy; ^2^Neuroscience Institute Cavalieri Ottolenghi (NICO), University of Torino, Turin, Italy; ^3^Computational Epidemiology Group and Data Analysis Unit, Department of Veterinary Sciences, University of Torino, Turin, Italy; ^4^Italian Institute for Genomic Medicine (IIGM), Turin, Italy; ^5^Department of Molecular Biotechnology and Health Sciences (MBC), University of Torino, Turin, Italy; ^6^Center for Translational Genomics and Bioinformatics, San Raffaele Scientific Institute (IRCCS), Milan, Italy

**Keywords:** Neuregulin1, Schwann cells, peripheral nerve injury, RNA deep sequencing, de-differentiation, demyelination, transcription, genetic

## Abstract

Peripheral nerves are characterised by the ability to regenerate after injury. Schwann cell activity is fundamental for all steps of peripheral nerve regeneration: immediately after injury they de-differentiate, remove myelin debris, proliferate and repopulate the injured nerve. Soluble Neuregulin1 (NRG1) is a growth factor that is strongly up-regulated and released by Schwann cells immediately after nerve injury. To identify the genes regulated in Schwann cells by soluble NRG1, we performed deep RNA sequencing to generate a transcriptome database and identify all the genes regulated following 6 h stimulation of primary adult rat Schwann cells with soluble recombinant NRG1. Interestingly, the gene ontology analysis of the transcriptome reveals that NRG1 regulates genes belonging to categories that are regulated in the peripheral nerve immediately after an injury. In particular, NRG1 strongly inhibits the expression of genes involved in myelination and in glial cell differentiation, suggesting that NRG1 might be involved in the de-differentiation (or “trans-differentiation”) process of Schwann cells from a myelinating to a repair phenotype. Moreover, NRG1 inhibits genes involved in the apoptotic process, and up-regulates genes positively regulating the ribosomal RNA processing, thus suggesting that NRG1 might promote cell survival and stimulate new protein expression. This *in vitro* transcriptome analysis demonstrates that in Schwann cells NRG1 drives the expression of several genes which partially overlap with genes regulated *in vivo* after peripheral nerve injury, underlying the pivotal role of NRG1 in the first steps of the nerve regeneration process.

## Introduction

Schwann cells represent the glial component of the peripheral nervous system. As well as supporting axonal function in a physiological status, they play a critical role during the degenerative and the regenerative processes activated after nerve injury (Namgung, 2014; Gordon, 2016; Jessen and Mirsky, 2016). A severe damage to the nerve tissue determines the loss of axon-Schwann cell contact with subsequent change in Schwann cell phenotype. Schwann cell de-differentiation occurs within 48 h after nerve injury and is driven by changes in gene expression with the down-regulation of genes related to myelination and node organisation, and the up-regulation of regeneration associated genes (RAG), such as growth factor receptors and adhesion molecules (Pereira et al., 2012; Jessen and Mirsky, 2016). De-differentiated Schwann cells isolate lipid droplets, composed by myelin, and participate to their degradation in the distal nerve. Few days after injury they proliferate and align to form the Büngner bands, a tubular structure that physically supports re-growing axons (Fawcett and Keynes, 1990; Ide, 1996). Moreover, Schwann cells are the source of growth factors that stimulate axon growth and create the appropriate supportive environment for axonal elongation (Hopker et al., 1999; Madl and Heilshorn, 2015). In the later phase of the regenerative process, they differentiate into myelinating and non-myelinating Schwann cells, determining the recovery of nerve morphology.

Schwann cell de-differentiation, proliferation and survival are regulated by the action of several growth factors. Among them, Neuregulin 1 (NRG1) increasingly attracted the attention for its involvement in several aspects of Schwann cell behaviour. NRG1 is a growth factor characterised by multiple isoforms thanks to alternative splicing (Mei and Xiong, 2008). Both soluble and transmembrane isoforms are present in peripheral nerves: transmembrane isoforms are expressed by neurons and are involved in myelin thickness regulation (Michailov et al., 2004; Taveggia et al., 2005), whereas soluble NRG1 isoforms are released both by Schwann cells and neurons and are thought to control Schwann cell survival, migration, de-differentiation, as well as the myelination process (Birchmeier and Nave, 2008; Fricker and Bennett, 2011; Stassart et al., 2013). NRG1 is strongly and transiently up-regulated in the distal stump immediately after nerve injury (Carroll et al., 1997; Stassart et al., 2013; Ronchi et al., 2016), suggesting a major role played by it in the response to nerve injury and in the nerve regeneration process.

Despite cellular effects induced by NRG1 stimulation, such as cell survival and migration, have been widely investigated (Dong et al., 1995; Zanazzi et al., 2001; Atanasoski et al., 2006; Birchmeier and Nave, 2008; Wakatsuki et al., 2014) and single genes have been demonstrated to be regulated by NRG1 in Schwann cells (Freidin et al., 2009; Iruarrizaga-Lejarreta et al., 2012; Sonnenberg-Riethmacher et al., 2015), a deep sequencing analysis of the genes modulated by NRG1 a few hours after stimulation has not yet been performed. Here, we present a global transcriptome analysis, aimed to identify the genes regulated *in vitro* by soluble NRG1 stimulation in primary rat Schwann cell culture.

We chose to analyse the transcriptome 6 h after NRG1 stimulation, to detect the early regulated genes and compare their expression pattern with the genes regulated *in vivo* after injury, where soluble NRG1 release and transcription are induced soon (Carroll et al., 1997; Guertin et al., 2005; Stassart et al., 2013; Ronchi et al., 2016; Yu et al., 2016) and a strong gene expression regulation is detectable between 6 h and 24 h (Yi et al., 2017).

## Materials and Methods

### Schwann Cell Primary Culture

To obtain Schwann cell primary cultures, sciatic nerves from adult female Wistar rats (ENVIGO, Milan, Italy) were isolated and harvested. This study was carried out in accordance with the recommendations of the Council Directive of the European Communities (2010/63/EU), the National Institutes of Health guidelines, and the Italian Law for Care and Use of Experimental Animals (DL26/14). The protocol was approved by the Italian Ministry of Health and the Bioethical Committee of the University of Torino. Conformed measures were taken into account to reduce the number of animals used and to minimise animal pain and discomfort.

Schwann cells from sciatic nerves were purified and cultured as previously described (Gnavi et al., 2015). Primary Schwann cells were routinely cultured on poly-L-lysine (PLL, Sigma)-coated plate, in complete medium consisting of DMEM (Sigma #D5671) supplemented with 10% heat-inactivated foetal bovine serum (FBS, Invitrogen), 100 units/ml penicillin, 0.1 mg/ml streptomycin, 1 mM sodium pyruvate, 2 mM L-glutamine, 8 nM recombinant soluble NRG1β1 (#396-HB, R&D Systems), 10 μM forskolin (Sigma) and incubated at 37°C in 5% CO_2_.

Schwann cells were cultured in the presence of 10 μM forskolin, because Schwann cell primary cultures display dedifferentiated cell features, having lost their axonal contact (Morrissey et al., 1991), but they can be induced to reacquire the differentiated phenotype (i.e., high myelin gene expression) by exposure to agents increasing the intracellular levels of cAMP (Sobue et al., 1986).

### Schwann Cell Stimulation and RNA Isolation

Confluent Schwann cells were starved overnight in starving medium consisting of DMEM (Sigma #D5671) supplemented with 2% heat-inactivated FBS, 100 units/ml penicillin, 0.1 mg/ml streptomycin, 1 mM sodium pyruvate, 2 mM L-glutamine, and 10 μM forskolin and then stimulated for 6 h with 10 nM recombinant soluble NRG1β1 (#396-HB, R&D Systems). Control mock samples were stimulated with the same volume of ligand resuspension buffer (PBS containing 1% bovine serum albumin/BSA, Sigma). After the stimulation, total RNA was isolated using TRIzol reagent (Invitrogen), following manufacturer’s instructions.

Schwann cell stimulation was performed in biological triplicate for deep sequencing analysis and in biological triplicate for gene expression validation through quantitative real time PCR analysis.

Biological triplicates were carried out using independent preparation of cells.

### Deep RNA Sequencing

Deep RNA sequencing was performed on three mock samples and three stimulated samples obtained in three independent experiments. RNA quality was assessed on an Agilent 2100 Bioanalyzer. All samples had RIN ≥9. For RNA-Seq library preparation, approximately 2 μg of total RNA were subjected to poly(A) selection and libraries were prepared using the TruSeq RNA Sample Prep Kit (Illumina) following the manufacturer’s instructions. Sequencing was performed on the Illumina NextSeq 500 platform.

The RNA Sequencing data have been deposited in the National Center for Biotechnology Information (NCBI) Gene Expression Omnibus (GEO), accessible through GEO Series accession number GSE104324.

### Analysis of RNA-Seq Data

Reads were mapped to the *Rattus norvegicus* rn5 reference assembly using TopHat v2.0.10 (Kim et al., 2013) and counts were generated using htseq and the refseq transcriptome. Genes with RPKM >1 in all three replicates were considered expressed in each condition. Differential expression was determined with DESeq2 (Love et al., 2014) with default parameters, implementing a paired design by using the biological replicate as covariate. Genes with adjusted *P* ≤ 0.01 and absolute log_2_ fold change >1 (fold change <0.5 and >2) were considered differentially expressed. Gene Ontology enrichment analysis was performed with ClusterProfiler (Yu et al., 2012) with a cutoff of *P* adjusted <0.1 (Supplementary Table S2). The analysis was performed using the gene ontology annotation of the mouse orthologs of our genes, due to the better functional annotation of the mouse genome.

### Analysis of Public Gene Expression Datasets

Two gene expression datasets, obtained from injured sciatic nerve samples, were down-loaded from GEO as normalised expression values.

#GSE22291 series corresponds to microcrushed adult mice sciatic nerves 3 and 7 days after injury, compared with control nerves; samples were hybridised to GeneChip Mouse Genome 430 2.0 Array (Affymetrix), platform GPL1261 (Barrette et al., 2010). Genes with adjusted *P* < 0.001 and absolute log_2_ fold change >1 were considered differentially expressed.

#GSE33454 series corresponds to cut adult mice sciatic nerves 1 and 5 days after injury, compared with sham control sciatic nerves; samples were analysed on Illumina MouseRef-8 v2.0 expression beadchip, platform GPL6885 (Kim et al., 2012). No biological replicates are available; genes with absolute log_2_ fold change >1 were considered differentially expressed.

Genes expressed 7 days after adult mice sciatic nerve cut, compared with contralateral uninjured nerves, were downloaded from the Supplementary Material of the article of Arthur-Farraj et al. (2017), where, to generate 100 bp paired-end reads, three libraries were run on a single lane of the HiSeq 2000 platform (Illumina). All selected genes have an adjusted *P* value < 0.05; those with an absolute log_2_ fold change >1 were considered differentially expressed.

Genes expressed 0.5 h, 1 h, 6 h, 12 h after adult mice sciatic nerve cut, compared with sham control sciatic nerves, were downloaded from the Supplementary Material of the article of Yi et al. (2017); samples were hybridised to Affymetrix GeneChip Hybridisation Oven 640. Genes with adjusted *P* < 0.05 and absolute log_2_ fold change >1 were considered differentially expressed.

### cDNA Preparation and Quantitative Real-Time PCR (qRT-PCR)

To validate the deep RNA sequencing analyses, 0.5 μg total RNA were retro-transcribed in a 25 μl reaction volume containing 1× RT buffer, 0.1 μg/μl BSA, 7.5 μM Random Hexamer Primers, 1 mM deoxynucleoside triphosphate (dNTPs), 0.05% Triton, 40U RIBOlock and 200U RevertAid^®^ Reverse Transcriptase (all ingredients were purchased by Thermo Scientific). The reaction was performed following the manufacturer’s instructions. qRT-PCR was performed by ABI Prism 7300 (Applied Biosystems, Life Technologies Europe BV) detection system. The cDNA was diluted 10 fold in nuclease-free water, and 5 μl (corresponding to 15 ng starting RNA) were analysed in a 20 μl reaction volume containing 1× iTaq Universal SYBR Green Supermix (BioRad) with 300 nM forward and 300 nM reverse primers. Dissociation curves were routinely performed to confirm the presence of a single peak corresponding to the required amplicon. Analyses were performed in technical and biological triplicate. For each experiment the average of mock samples was used as a calibrator. The threshold cycle (Ct) values were normalised to TATA Binding Protein (TBP), a general RNA polymerase II transcription factor, which has been shown to be a good housekeeping gene without retro-pseudogenes in the genome and with different exons to design forward and reverse primers separated by an intron (Vandesompele et al., 2002). Expression data were shown as −ΔΔCT to compare qRT-PCR results with deep sequencing data expressed as log_2_ (fold change). Primers were designed using Annhyb software^1^ and synthesised by BMR Genomics. Primer sequences are reported in Table 1.

**Table 1:** List of primers used for quantitative real time PCR (qRT-PCR) analysis to validate deep sequencing results.

Gene	Accession number	Forward primer (5′–3′)	Reverse primer (5′–3′)	Amplicon size
Atf3	NM_012912.2	CACCATCAACAACAGACCTCTGGAG	CCGCCGCCTCCTTTTTCTCTC	85 bp
Bmp7	NM_001191856.2	CGTCAACCTAGTGGAGCACGAC	GTCACCGCCTCTCCCTCGG	102 bp
Egr2	NM_053633.1	GACCATCTTCCCCAATGGTGAACTG	GATATGGGAGATCCAAGGGCCTCTTC	119 bp
Hmga2	NM_032070.1	CGCCACAGAAGCGAGGACG	GGGGCTCTTGTTCTTGCTGCC	113 bp
Inhba	NM_017128.2	CGGAGATCATCACCTTTGCCGAG	CAGGAAGAGCCAGACTTCTGCAC	116 bp
Mag	NM_017190.4	CGCCTTCAACCTGTCTGTGGAGT	GCCACGGAGGGTTCCGG	120 bp
Mbp	NM_017026.2	GGACCCAAGATGAAAACCCAGTAGTCC	CCTTTCCTTGGGATGGAGGGGG	81 bp
Pmp22	NM_017037.1	CCTTGGGAGCCGTCCAGC	GGACGCTGAAGATGACAGACAGGATC	69 bp
Shc4	NM_001191065.1	CACTTGGGAAAGGGAGGAGGTCC	CACATCTGCAATGCCGCCTG	112 bp
Tbp	NM_001004198.1	TAAGGCTGGAAGGCCTTGTG	TCCAGGAAATAATTCTGGCTCATAG	68 bp
Vegfc	NM_053653.1	CGTCGCCGCCTTCGAGTC	GCTCATCTACACTGGACACAGACCG	122 bp

### Statistical Analysis

For qRT-PCR data, statistical analysis between two groups (mock and treated samples) was performed in GraphPad Prism 5 by the two-tailed Student’s *t*-test. All data were expressed as mean + standard error (SEM).

## Results

### Several Genes in Schwann Cells Are Regulated By Soluble NRG1 Stimulation

To identify the genes regulated early by soluble NRG1, deep sequencing analysis was applied on RNA samples obtained from Schwann cells stimulated 6 h with 10 nM recombinant soluble NRG1β1 (from here on called NRG1) and mock stimulated cells, in three independent biological replicates. Preliminary assays had been previously performed to verify that treatment with 10 nM NRG1 is able to stimulate ErbB3, AKT, ERK phosphorylation (data not shown).

We identified the genes expressed in the biological triplicate by mock Schwann cells (11312, 10810, 11142) and by Schwann cells following NRG1 stimulation (11239, 10811, 11197). Comparing the NRG1 stimulated cells with the mock cells, 190 genes were significantly differentially expressed: 101 up-regulated, 89 down-regulated (with a |log_2_fold change|>1, corresponding to a fold change <0.5 and >2, with a P adjusted ≤0.01), with 93% of the differentially expressed genes having an adjusted *P* value < 0.001.

**Figure 1:**
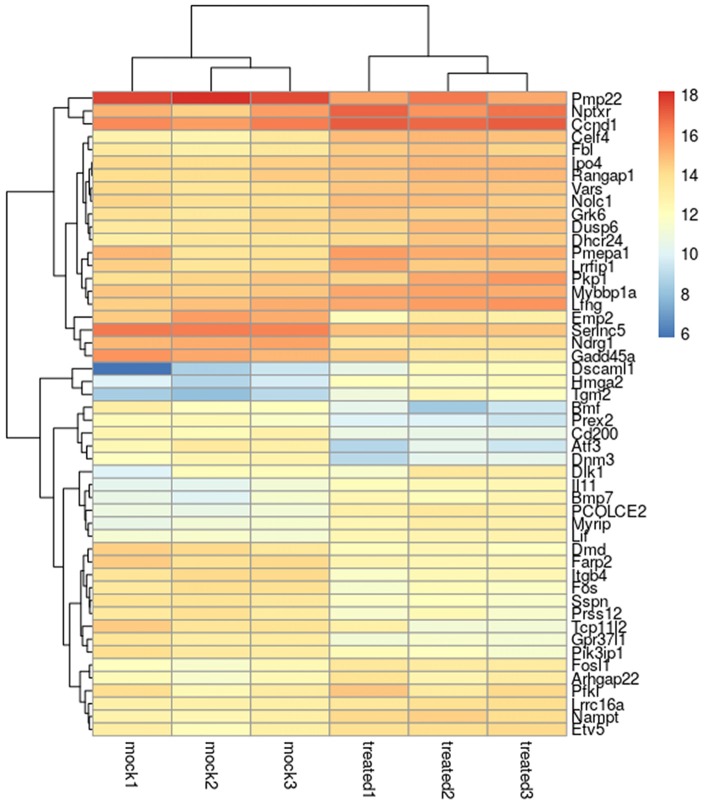
Heatmap of the 50 most differentially regulated genes in Schwann cells. The heatmap shows the biological triplicate expression of the first 50 differentially expressed genes in Schwann cells stimulated with 10 nM NRG1β1 based on the obtained *P* value. “Mock” corresponds to samples treated with the vehicle, “treated” corresponds to samples treated with NRG1β1. Colour intensity reflects the expression level and represents the log_2_ (RPKM + 1) of the observed gene in each sample (RPKM, Reads Per Kilo base of transcript per Million mapped reads).

A heat map of the 50 most differentially regulated genes is shown in Figure 1, while the full list of differentially expressed genes is shown in Supplementary Table S1.

To validate deep RNA sequencing data, the expression profile of 10 selected genes was analysed by quantitative real time PCR performed on RNA samples obtained from mock Schwann cells or stimulated 6 h with 10 nM NRG1. Validation was performed on three independent biological replicates different from those used for the deep sequencing.

**Figure 2:**
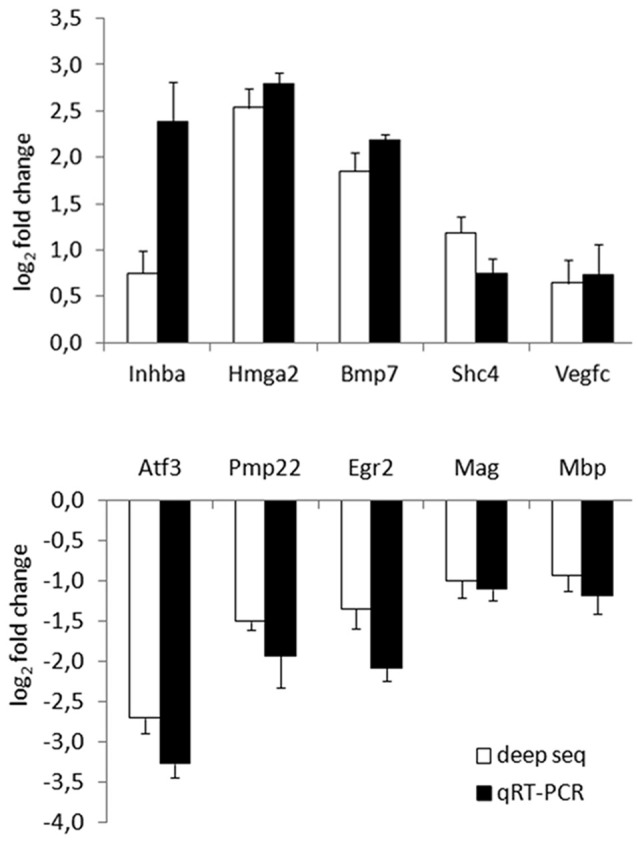
Deep RNA sequencing validation through quantitative real time-PCR (qRT-PCR). Validation of 10 representative gene transcripts was performed by qRT-PCR, showing that the behaviour of five up- and five down-regulated genes was consistent in both techniques. log_2_ fold change (corresponding to −ΔΔCT for qRT-PCR) obtained comparing samples treated with Neuregulin1 (NRG1) with mock treated samples is shown, both for deep sequencing, both for qRT-PCR data.

Five up- and five down-regulated genes were chosen based on their adjusted *P* values or their annotated Gene Ontology categories. Among them, eight were strongly regulated (corresponding to 4.2% of differentially regulated genes), two weakly; all of them were significantly validated. Inhba, Hmga2, Bmp7, Shc4, Vegfc were representative for up-regulated genes, Atf3, Pmp22, Egr2, Mag, Mbp were representatives for down-regulated genes. As shown in Figure 2, the expression profile of these genes was consistent in both analyses.

### NRG1 Strongly Down-Regulates Myelination Genes

In order to assign the differentially regulated genes to their functions, Gene Ontology analysis was performed using ClusterProfiler package, focusing the attention on the biological processes (BP).

Genes up-regulated following NRG1 stimulation were enriched in two annotation categories: 11 genes belong to the category of “rRNA metabolic processes” (*P*_adj_ = 0.0001), four genes belong to “negative regulation of Notch signalling pathway” (*P*_adj_ = 0.0219); the complete list of these up-regulated genes is shown in Table 2.

**Table 2:** Biological process (BP) enriched categories obtained by Gene Ontology analysis.

Biological process enriched category	Down-regulated genes	*P* adjusted	List of genes
Myelination	7	0.004263845	Pmp22, Serinc5, Ndrg1, Fa2 h, Mal, Rxrg, Egr2
Positive regulation of ion transmembrane transport	6	0.006544373	Dmd, Hspa2, Snca, P2rx7, Atp1b2, Hcn1
Glial cell differentiation	8	0.008986003	Gpr37l1, Ndrg1, Dmd, Epha4, Fa2 h, Egr2, Ntrk3, Lingo1
Regulation of membrane potential	9	0.010459252	Dmd, Snca, P2rx7, Grik2, Atp1b2, Kcnk5, Cldn19, Slc26a2, Hcn1
Bleb assembly	3	0.023620351	Emp2, Pmp22, P2rx7
Positive regulation of apoptotic process	11	0.080348376	Atf3, Bmf, Gadd45a, Ctsc, Snca, P2rx7, Grik2, Mal, Fbxo32, Ntrk3, Sept4
Macrophage activation	3	0.095570035	Cd200, Snca, Adgrf5
**Biological process enriched category**	**Up-regulated genes**	***P* adjusted**	**List of genes**
rRNA metabolic processes	11	0.000126	Fbl, Bop1, Wdr46, Nop56, Ddx21, Nat10, Bysl, Rrp9, Nop2, Emg1, Rrp15
Negative regulation of Notch signalling pathway	4	0.02191	Bmp7, Dlk1, Lfng, Dll4

*Because of redundancy in gene ontology annotations, only a selected list of up-regulated and down-regulated biological processes is shown (all enriched categories of biological processes, molecular functions and cellular components are shown in Supplementary Table S2)*.

Due to redundancy in gene ontology annotations, here we show a selected list of up-regulated and down-regulated BP; the complete list of BP, Molecular Functions (MF) and Cellular Components (CC) is shown in Supplementary Table S2.

Functional enrichment is stronger for down-regulated genes. “Myelination” (*P*_adj_ = 0.0043) is the most enriched category among down-regulated genes, with seven genes down-regulated in response to NRG1. The following categories are “Positive regulation of ion transmembrane transport” (*P*_adj_ = 0.0065) with six regulated genes, “Glial cell differentiation” (*P*_adj_ = 0.0090) with eight genes, “Regulation of membrane potential” (*P*_adj_ = 0.0105) with nine genes, “Bleb assembly” with three genes (*P*_adj_ = 0.0236). Finally, “Positive regulation of apoptotic process” (*P*_adj_ = 0.0803) with 11 genes, “Macrophage activation” (*P*_adj_ = 0.0956) with three. The complete list of these down-regulated genes is shown in Table 2.

### Several Genes Regulated By NRG1 Overlap With Genes Regulated Following Nerve Injury

As expected, the genes regulated by NRG1 are annotated in categories that are related to Schwann cell response following an injury. To identify which of these genes are also regulated after a nerve injury, expression profiling data obtained from injured sciatic nerve samples were downloaded from GEO or from Supplementary Information of published articles.

**Figure 3:**
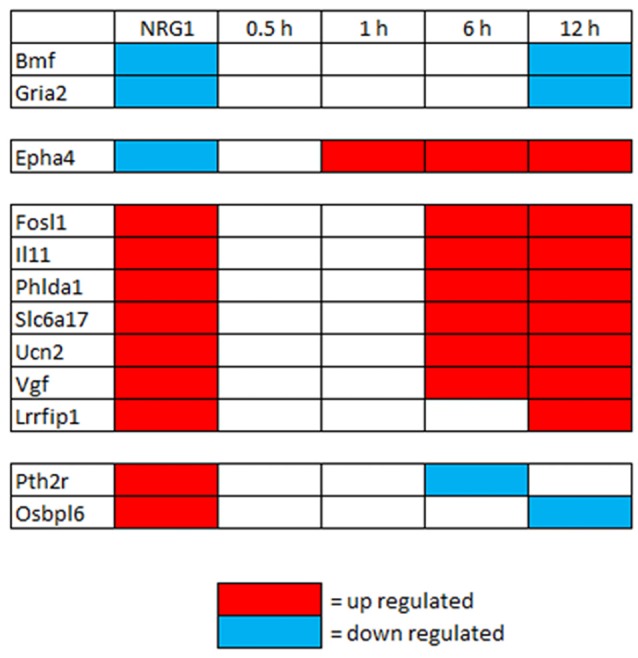
Genes differentially regulated *in vitro* by NRG1 compared with 0.5–12 h injured nerves. Genes significantly regulated following NRG1 stimulation (101 up- and 89 down-) were compared with genes regulated 0.5 h, 1 h, 6 h, and 12 h after sciatic nerve cut (Yi et al., 2017). Among them, seven up-regulated and two down-regulated show the same regulation behaviour after 12 h.

To investigate gene expression in the first hours after injury, genes regulated by NRG1 (101 up- and 89 down-regulated) were compared with genes differentially expressed 0.5 h, 1 h, 6 h, 12 h after nerve cut (Yi et al., 2017). This analysis shows that after 0.5 and 1 h there are not differentially expressed genes in common with our data. After 6 h, six up-regulated genes are in common, after 12 h, seven up-regulated and two down-regulated genes are in common with our data (Figure 3).

**Figure 4:**
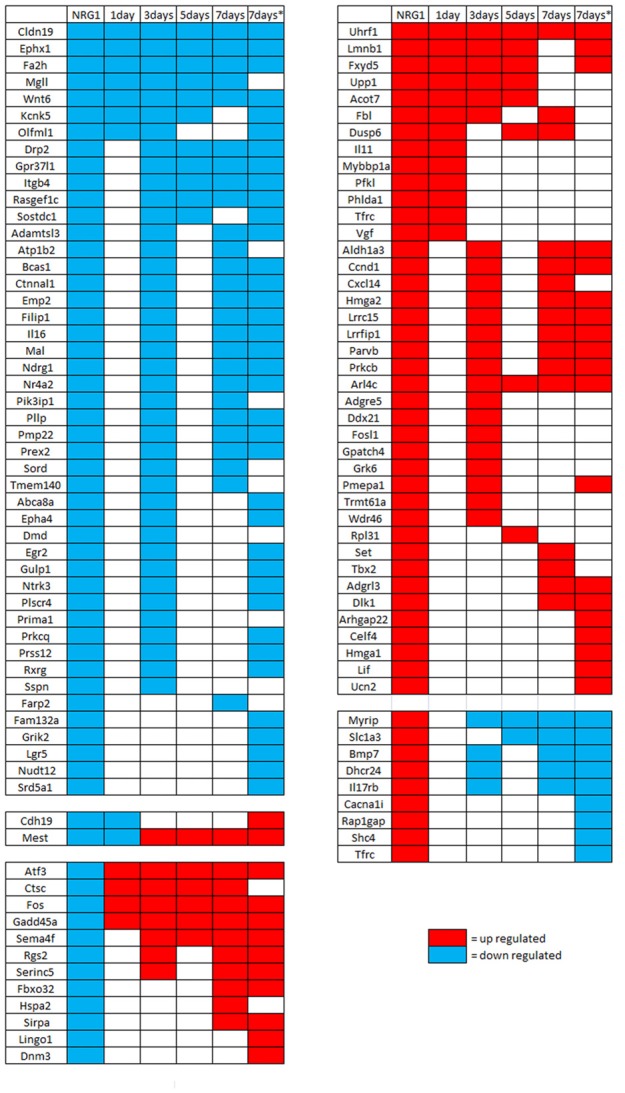
Genes differentially regulated *in vitro* by NRG1 compared with 1–7 days injured nerves. Genes significantly regulated following NRG1 stimulation (101 up- and 89 down-) were compared with genes regulated 1 and 5 days after sciatic nerve cut (Kim et al., 2012), 3 and 7 days after sciatic nerve microcrush (Barrette et al., 2010), and 7 days (*) after sciatic nerve cut (Arthur-Farraj et al., 2017). Among them, 40 up-regulated and 46 down-regulated show the same regulation behaviour at different time points after injury, 9 up-regulated and 12 down-regulated show an opposite behaviour, two down-regulated genes show a mixed behaviour.

Then, to investigate gene expression in the following phase after injury, genes regulated by NRG1 were compared with genes regulated 1 and 5 days after nerve cut (Kim et al., 2012), 3 and 7 days after nerve microcrush (Barrette et al., 2010), 7 days after nerve cut (Arthur-Farraj et al., 2017). These data show that in the first week of injury 54% of NRG1 down-regulated genes and 40% of NRG1 up-regulated genes are also regulated after nerve injury: 13 up-regulated and 9 down-regulated 1 day after nerve cut; 23 up-regulated and 40 down-regulated 3 days after nerve microcrush; 8 up-regulated and 11 down-regulated 5 days after nerve cut; 16 up-regulated and 26 down-regulated 7 days after nerve microcrush. Many genes show the same regulation at different time points. Intriguingly, few genes (9 up- and 12 down-regulated) show an opposite expression regulation, two genes are down-regulated 1 day after injury and after NRG1 treatment, but later their expression is up-regulated. The complete list of genes regulated both by NRG1 and by nerve injury is shown in Figure 4.

## Discussion

Schwann cells, the main glial cells in the peripheral nervous system, play a key role both during development and following nerve injury, being responsible of axonal ensheathment, myelination and post-injury nerve repair (Jessen et al., 2015; Taveggia, 2016). Different factors and signalling pathways are involved in the control of these activities (Pereira et al., 2012; Boerboom et al., 2017).

It has been shown by others (Carroll et al., 1997; Stassart et al., 2013) and our research group (Ronchi et al., 2016) that different isoforms of soluble NRG1 are strongly up-regulated after injury. Moreover, in transgenic mice expressing soluble NRG1 in postnatal motoneurons and DRG neurons, but missing NRG1 in Schwann cells (Thy1.2-NRG1-I-β1a X Dhh-Cre X Nrg1^loxP/loxP^), nerve regeneration after crush is inefficient (Stassart et al., 2013), thus showing that soluble Nrg1 expressed by neurons does not completely compensate for the lack of NRG1 expression in Schwann cells, thus suggesting that soluble NRG1 plays a key role for their activity after injury.

Schwann cells represent 90% of the total cell number in uncut nerves, 70% in injured nerves, the majority of the rest being fibroblasts, macrophages and perineural cells (Arthur-Farraj et al., 2017). The role of the latter must not be denied, but here we focused our attention on Schwann cells, the main non-neuronal cell type in the peripheral nerve, to investigate the role of soluble NRG1 in their activity.

To this aim, by RNA deep sequencing, we carried out a transcriptome analysis to identify the genes regulated *in vitro* in Schwann cells following NRG1 stimulation.

Then, we compared this list of differentially regulated genes with the genes expressed *in vivo* in the injured nerve to find common regulated genes, conscious of the fact that in the injured nerve environment, besides Schwann cells, there are other different cell types, and that there are other released factors besides soluble NRG1.

A stringent analysis was performed to select genes significantly regulated *in vitro* by NRG1 stimulation with a 2-fold threshold and a highly significant adjusted *P* value (≤0.01). As expected, NRG1 stimulation induces differential gene expression in Schwann cells. Applying gene ontology, the regulated genes were categorised into specific groups based on the relevant BP, CC and MF. We focused our attention on the BP; after eliminating redundant categories, down-regulated genes were enriched in seven categories, while up-regulated genes were enriched in two.

Enriched gene categories are consistent with the phenotypic changes occurring in Schwann cells after injury: the portion of the nerve distal to the injury undergoes Wallerian degeneration, a complex process characterised by axonal degeneration and degradation of myelin sheath, followed by debris phagocytosis by Schwann cells and macrophages (Dubovy, 2011). Demyelination is an important step during Wallerian degeneration, starting with fragmentation of the myelin sheath into small ovoid-like structures (Ghabriel and Allt, 1979) and with the down-regulation of myelin genes (Jessen and Mirsky, 2008). Because NRG1 receptor activation is sufficient to initiate Schwann cell demyelination (Guertin et al., 2005), it has been suggested that NRG1 might play a key role in the de-differentiation process (Fricker and Bennett, 2011; Shin et al., 2013).

Both myelinating and non-myelinating (Remak) Schwann cells participate to Wallerian degeneration and trans-differentiate into a repair phenotype specialised to promote nerve regeneration (Jessen and Mirsky, 2016). Accordingly, when we consider gene expression analysis *in vivo*, both myelinating and non-myelinating Schwann cells contribute to the transcriptome, as well as *in vitro*, where primary cultures obtained from sciatic nerves contain both myelinating and non-myelinating cells.

When we analysed the enriched categories of differentially expressed genes in response to NRG1 stimulation, we found that “myelination” is the most enriched category among down-regulated genes, with several genes known to be involved in the myelination process down-regulated (Pmp22, Serinc5, Ndrg1, Fa2h, Mal, Rxrg and Krox20/Egr2), thus suggesting that myelination genes were expressed by myelinating Schwann cells undergoing trans-differentiation, while the enriched down-regulated category “glial cell differentiation” reflects gene expression of both myelinating and non-myelinating Schwann cells.

The fact that NRG1 inhibits myelination genes evokes literature data showing that the transcription factor c-Jun negatively regulates myelination (Parkinson et al., 2008), down-regulating genes essential in the myelination program, such as the zinc finger transcription factor Krox20/Egr2 (Warner et al., 1998), which in turn has been shown to inhibit c-Jun expression in a reciprocal negative feed-back loop (Parkinson et al., 2004).

We found that also soluble NRG1 inhibits Krox20/Egr2 expression, thus suggesting that NRG1—inhibiting Krox20/Egr2—might contribute to stimulate c-Jun expression. Accordingly, it has been shown *in vitro* that NRG1 treatment stimulates c-Jun expression (Syed et al., 2010) and phosphorylation (Parkinson et al., 2004). Further analyses *in vitro* and *in vivo* will be necessary to better investigate the cross-talk between c-Jun and NRG1.

Genes annotated in categories like “positive regulation of apoptotic process” and “bleb assembly” are also down-regulated, suggesting that NRG1 supports Schwann cell survival following nerve injury.

NRG1 stimulation of Schwann cells showed a down-regulation of genes annotated in the enriched category “regulation of macrophage activation”, such as Cd200 and Snca. Macrophages are recruited to the nerve injured site to eliminate fragmented myelin debris and dead cells (Dubovy, 2011). Cd200 is expressed in node of Ranvier and Schmidt-Lanterman incisures and is down-regulated following nerve injury (Chang et al., 2011). It has been suggested that Cd200 down-regulation might facilitate macrophage infiltration through the node of Ranvier to eliminate degenerated axons and myelin debris (Chang et al., 2011). Also Synuclein α (Snca) is localised at nodes of Ranvier and Schmidt-Lanterman incisures, is up-regulated during myelination and down-regulated after nerve injury (D’Antonio et al., 2006) and might be involved in macrophage infiltration. Indeed, it has been shown that following sciatic nerve transection, transgenic mice over-expressing Snca show a lower number of invading macrophages (Siebert et al., 2010).

Two categories of up-regulated genes are enriched in response to NRG1 stimulation: “genes responsible for rRNA metabolic processes” and “negative regulation of Notch signalling pathway”. The up-regulation of the former contributes to the synthesis and production of new proteins and molecules needed to guide Wallerian degeneration and nerve regeneration.

Notch is a transmembrane receptor that acts as a negative regulator of myelination: it is down-regulated during myelination and by Krox20, and plays an important role in demyelination in injured nerves (Mirsky et al., 2008; Woodhoo et al., 2009). For this reason, the up-regulation of negative regulators of the Notch pathway is difficult to explain, unless we hypothesise that Notch is up-regulated in a different time point or that Notch expression is regulated at protein level, or that the genes grouped in this gene ontology category actually contribute to demyelination and Schwann cell de-differentiation without inhibiting Notch pathway. Indeed, Bmp7 retards peripheral myelination by activating p38 MAPK in Schwann cells (Liu et al., 2016), while delta like non-canonical Notch ligand 1 (Dlk1) and Delta-like 4 Notch ligand (Dll4) are both Notch ligands.

Since NRG1 is strongly up-regulated after injury and Schwann cells represent a high percentage of cells in the injured nerve, we hypothesised that their contribution to the injured nerve transcriptome is relevant. Therefore, we compared the list of genes regulated in Schwann cells in response to NRG1 stimulation with the lists of genes regulated after nerve injury, with the idea to identify, among many regulated genes, the genes regulated in Schwann cells following NRG1 stimulation.

To this aim, we exploited previously published expression profiling data of injured sciatic nerves at different early time points (0.5, 1, 6, 12 h and 1, 3, 5, 7 days) following injury (Barrette et al., 2010; Kim et al., 2012; Yi et al., 2017). These data were obtained using microarray assay; more recently, deep sequencing data at 7 days after injury were published and made available to the scientific community (Arthur-Farraj et al., 2017). Thus, in Figure 4, we show two data set corresponding to 7 days after injury; they do not correspond 100%, likely because the first set derives from a microcrushed nerve, the second from a transected nerve; moreover, the first was analysed by microarray, the second was analysed by deep sequencing. Accordingly, the authors of the latter analysis compared their deep sequencing analysis with a previous microarray analysis (Arthur-Farraj et al., 2012), finding that ~20% of regulated genes were differently regulated. As expected, several genes that are regulated by NRG1 in Schwann cells overlap with genes regulated in the sciatic nerve following nerve injury. Nevertheless, some genes show a different regulation. These differences can be explained by the fact that genes expressed in injured sciatic nerves belong mainly to Schwann cells, but also to other cell types, while the gene expression profile obtained with the deep sequencing corresponds to genes expressed only by Schwann cells. Moreover, in the injured nerve environment several factors are released, not only soluble NRG1, and the nerve analysis reveals genes regulated by different factors.

*Ex vivo* primary cultures represent a good experimental model as they allow focusing the analysis on a single cell type (e.g., Schwann cells), while *in vivo* different cell types coexist in the injured nerve: Schwann cells, fibroblasts, macrophages, epineural cells, and so on. Moreover, they allow analysing a single factor stimulation (e.g., NRG1), while *in vivo* different factors are released after injury.

Our* in vitro* analysis focused on genes regulated after 6 h stimulation; we chose this time point because we were interested to identify genes regulated early by NRG1 in Schwann cells. Indeed, *in vivo* experiments showed that after nerve injury NRG1 mRNA increase is detectable between 1 h and 24 h (Carroll et al., 1997; Stassart et al., 2013; Ronchi et al., 2016; Yu et al., 2016) and several genes start to be differentially expressed between 6 h and 24 h (Yi et al., 2017).

By comparing the differentially expressed genes in Schwann cells after *in vitro* NRG1 stimulation with differentially expressed genes in peripheral nerves in response to injury, we found that many genes show the same behaviour in the two experimental models (40% up- and 54% down-regulated) while only 11% show an opposite behaviour. As previously discussed, this opposite behaviour might be explained by the fact that the expression of these genes in the peripheral nerve is not regulated in Schwann cells, but rather in other cell types belonging to the nerve tissue, or by the fact that the expression of these genes in injured Schwann cells is also regulated by other factors released in the injured environment. Among them, we noticed the transcription factors Atf3 and Fos (Patodia and Raivich, 2012) and Gadd45a (Lin et al., 2011), which are significantly down-regulated by NRG1 in Schwann cells *in vitro*, while *in vivo* are up-regulated in sciatic nerves (Arthur-Farraj et al., 2017) and dorsal root ganglia (DRG) neurons after injury.

First, we compared genes differentially expressed *in vitro* in NRG1 stimulated Schwann cells with genes differentially expressed *in vivo* a few hours after nerve injury (0.5–12 h). In the early hours we found more up-regulated than down-regulated common genes. Four of them (Il11, Phlda1, Slc6a17, Vgf) are up-regulated in the first 12–24 h only, thus suggesting that they may play a role immediately after injury.

Then, we focused our attention on up-regulated and down-regulated genes common to all time points (from day 1 until day 7) following nerve injury and following Schwann cell NRG1 stimulation. In this time window, we found more down-regulated than up-regulated common genes.

A common up-regulated gene is Ubiquitin Like with PHD and Ring Finger Domains 1 (Uhrf1), encoding for a E3 ubiquitin ligase. This protein binds to specific DNA sequences, regulating gene expression. It has been shown that Uhrf1 plays a role in inducing basal cell proliferation following airway injury (Xiang et al., 2017), thus suggesting that it might play a similar role in stimulating proliferation of Schwann cells following a peripheral nerve injury.

Another common up-regulated gene is Lamin B1 (Lmnb1), playing an important role in the modulation of genes involved in normal myelin regulation; moreover, Lmnb1 over-expression is associated with down-regulation of proteolipid protein, a highly abundant myelin sheath component (Heng et al., 2013). Intriguingly, another gene regulated at all time points after injury and after NRG1 stimulation is FXYD domain containing ion transport regulator 5 (Fxyd5), which has been recently shown to be a regeneration-associated gene (RAG), whose over-expression increases (and knock-down decreases) neuritis length and number (Chandran et al., 2016).

The common down-regulated genes include Cldn19, Ephx1, Wnt6 and Fa2h.

Tight-Junction Gene Claudin 19 (Cldn19) is the main tight junction constituent of Schwann cells and it is involved in their electrophysiological sealing function (Miyamoto et al., 2005) in the healthy nerve. Microsomal epoxide hydrolase (Ephx1) is an evolutionarily highly conserved enzyme expressed in nearly all tissues, where it can play a role in redox homeostasis by removing reactive oxygen species (Václavíková et al., 2015). Abnormalities in Ephx1 expression are involved in neurological disorders such as Alzheimer’s disease (Liu et al., 2006) and tumours. As far as we know, nothing was mentioned in literature about its role in peripheral nerves or Schwann cells.

Wnt is expressed by axons, and it has been found that Wnt signalling promotes Schwann cell lineage progression, proliferation and myelination (Grigoryan et al., 2013).

Fatty acid 2-hydroxylase (Fa2h), which is down-regulated from the first till the 7th day post injury, is found to be directly related to the process of myelin formation, where it is required for the synthesis of 2-hydroxy galactolipids in peripheral nerve myelin (Maldonado et al., 2008).

Other genes that are down-regulated by NRG1 and related to the myelination process (Mal, Ndrg1, Pmp22, Egr2, Rxrg) are also down-regulated at different time points after injury, suggesting that NRG1 might be involved in the demyelination process following nerve injury.

The down-regulation of genes involved in the myelination is particularly intriguing in the light of our recent data obtained from a rat model of the demyelinating neuropathy Charcot-Marie-Tooth 1A, where we demonstrated that soluble NRG1 is strongly over-expressed in the nerves (Fornasari et al., 2018). We wondered if soluble NRG1 over-expression worsens the disease or counteracts it, attenuating its symptoms and promoting nerve repair. The down-regulation of myelination genes suggests that it worsens the disease and that therapeutic approaches aimed to inhibit NRG1 activity could be more useful than therapeutic approaches involving treatment with soluble NRG1 (Fledrich et al., 2014). Accordingly, it has been shown that a high concentration of soluble NRG1 negatively affects *in vitro* myelination (Syed et al., 2010).

Our *in vitro* analysis shows that soluble NRG1 plays a key role for Schwann cell survival, de-differentiation and demyelination, thus suggesting that *in vivo*, after peripheral nerve injury, soluble NRG1 release or delivery needs to be limited to the early phases after nerve injury. Indeed, in the following phases, the remyelination is promoted by the NRG1 transmembrane isoforms expressed by the axons (Michailov et al., 2004; Taveggia et al., 2005).

This concept must be kept in mind when planning gene therapies with soluble NRG1 or recombinant factor delivery aimed to promote peripheral nerve regeneration.

## Author Contributions

MES and GG designed the study, prepared the figures and drafted the manuscript. MES prepared primers and validated deep sequencing data by quantitative real time PCR. MES, BEF, MM, GR and GG prepared primary Schwann cells, stimulated them with NRG1 and extracted RNA. DI performed deep sequencing analysis. EG, MG and PP performed deep sequencing data analysis, statistical analysis and gene ontology. SG and PP critically revised the manuscript.

## Conflict of Interest Statement

The authors declare that the research was conducted in the absence of any commercial or financial relationships that could be construed as a potential conflict of interest.

## Footnotes

^1^http://www.bioinformatics.org/annhyb/

## Supplementary Material

TABLE S1 Differential expression analysis. Sheet 1: deseq2 results for all genes; Sheets 2 and 3: up- and down-regulated genes (adjusted *P* < 0.05 and fold-change >2 or <0.5).

TABLE S2 The complete list of enriched categories of Biological Processes (BP), Molecular Functions (MF) and Cellular Components (CC) obtained by Gene Ontology enrichment analysis of regulated genes.

## References

[B1] Arthur-Farraj P. J. Latouche M. Wilton D. K. Quintes S. Chabrol E. Banerjee A.. (2012). c-Jun reprograms Schwann cells of injured nerves to generate a repair cell essential for regeneration. Neuron 75, 633–647. 10.1016/j.neuron.2012.06.021 22920255 PMC3657176

[B2] Arthur-Farraj P. J. Morgan C. C. Adamowicz M. Gomez-Sanchez J. A. Fazal S. V. Beucher A.. (2017). Changes in the coding and non-coding transcriptome and DNA methylome that define the schwann cell repair phenotype after nerve injury. Cell Rep. 20, 2719–2734. 10.1016/j.celrep.2017.08.064 28903050 PMC5608958

[B3] Atanasoski S. Scherer S. S. Sirkowski E. Leone D. Garratt A. N. Birchmeier C.. (2006). ErbB2 signaling in Schwann cells is mostly dispensable for maintenance of myelinated peripheral nerves and proliferation of adult Schwann cells after injury. J. Neurosci. 26, 2124–2131. 10.1523/JNEUROSCI.4594-05.2006 16481445 PMC6674935

[B4] Barrette B. Calvo E. Vallieres N. Lacroix S. (2010). Transcriptional profiling of the injured sciatic nerve of mice carrying the Wld(S) mutant gene: identification of genes involved in neuroprotection, neuroinflammation, and nerve regeneration. Brain Behav. Immun. 24, 1254–1267. 10.1016/j.bbi.2010.07.249 20688153

[B5] Birchmeier C. Nave K. A. (2008). Neuregulin-1, a key axonal signal that drives Schwann cell growth and differentiation. Glia 56, 1491–1497. 10.1002/glia.20753 18803318

[B6] Boerboom A. Dion V. Chariot A. Franzen R. (2017). Molecular mechanisms involved in schwann cell plasticity. Front. Mol. Neurosci. 10:38. 10.3389/fnmol.2017.00038 28261057 PMC5314106

[B7] Carroll S. L. Miller M. L. Frohnert P. W. Kim S. S. Corbett J. A. (1997). Expression of neuregulins and their putative receptors, ErbB2 and ErbB3, is induced during Wallerian degeneration. J. Neurosci. 17, 1642–1659. 10.1523/JNEUROSCI.17-05-01642.1997 9030624 PMC6573392

[B8] Chandran V. Coppola G. Nawabi H. Omura T. Versano R. Huebner E. A.. (2016). A systems-level analysis of the peripheral nerve intrinsic axonal growth program. Neuron 89, 956–970. 10.1016/j.neuron.2016.01.034 26898779 PMC4790095

[B9] Chang C. Y. Lee Y. H. Jiang-Shieh Y. F. Chien H. F. Pai M. H. Chen H. M.. (2011). Novel distribution of cluster of differentiation 200 adhesion molecule in glial cells of the peripheral nervous system of rats and its modulation after nerve injury. Neuroscience 183, 32–46. 10.1016/j.neuroscience.2011.03.049 21453758

[B10] D’Antonio M. Michalovich D. Paterson M. Droggiti A. Woodhoo A. Mirsky R.. (2006). Gene profiling and bioinformatic analysis of Schwann cell embryonic development and myelination. Glia 53, 501–515. 10.1002/glia.20309 16369933

[B11] Dong Z. Brennan A. Liu N. Yarden Y. Lefkowitz G. Mirsky R.. (1995). Neu differentiation factor is a neuron-glia signal and regulates survival, proliferation and maturation of rat Schwann cell precursors. Neuron 15, 585–596. 10.1016/0896-6273(95)90147-7 7546738

[B12] Dubovy P. (2011). Wallerian degeneration and peripheral nerve conditions for both axonal regeneration and neuropathic pain induction. Ann. Anat. 193, 267–275. 10.1016/j.aanat.2011.02.011 21458249

[B13] Fawcett J. W. Keynes R. J. (1990). Peripheral nerve regeneration. Annu. Rev. Neurosci. 13, 43–60. 10.1146/annurev.ne.13.030190.000355 2183684

[B14] Fledrich R. Stassart R. M. Klink A. Rasch L. M. Prukop T. Haag L.. (2014). Soluble neuregulin-1 modulates disease pathogenesis in rodent models of Charcot-Marie-Tooth disease 1A. Nat. Med. 20, 1055–1061. 10.1038/nm.3664 25150498

[B15] Fornasari B. E. Ronchi G. Pascal D. Visigalli D. Capodivento G. Nobbio L.. (2018). Soluble Neuregulin1 is strongly up-regulated in the rat model of Charcot-Marie-Tooth 1A disease. Exp. Biol. Med. 243, 370–374. 10.1177/1535370218754492 29350067 PMC6022927

[B16] Freidin M. Asche S. Bargiello T. A. Bennett M. V. Abrams C. K. (2009). Connexin 32 increases the proliferative response of Schwann cells to neuregulin-1 (Nrg1). Proc. Natl. Acad. Sci. U S A 106, 3567–3572. 10.1073/pnas.0813413106 19218461 PMC2651262

[B17] Fricker F. R. Bennett D. L. (2011). The role of neuregulin-1 in the response to nerve injury. Future Neurol. 6, 809–822. 10.2217/fnl.11.45 22121335 PMC3223410

[B18] Ghabriel M. N. Allt G. (1979). The role of Schmidt-Lanterman incisures in Wallerian degeneration. I. A quantitative teased fibre study. Acta Neuropathol. 48, 93–93. 10.1007/bf00691149 506700

[B19] Gnavi S. Fornasari B. E. Tonda-Turo C. Ciardelli G. Zanetti M. Geuna S.. (2015). The influence of electrospun fibre size on Schwann cell behaviour and axonal outgrowth. Mater. Sci. Eng. C Mater. Biol. Appl. 48, 620–631. 10.1016/j.msec.2014.12.055 25579965

[B20] Gordon T. (2016). Nerve regeneration in the peripheral and central nervous systems. J. Physiol. 594, 3517–3520. 10.1113/JP270898 27365158 PMC4929322

[B21] Grigoryan T. Stein S. Qi J. Wende H. Garratt A. N. Nave K. A.. (2013). Wnt/Rspondin/β-catenin signals control axonal sorting and lineage progression in Schwann cell development. Proc. Natl. Acad. Sci. U S A 110, 18174–18179. 10.1073/pnas.1310490110 24151333 PMC3831430

[B22] Guertin A. D. Zhang D. P. Mak K. S. Alberta J. A. Kim H. A. (2005). Microanatomy of axon/glial signaling during Wallerian degeneration. J. Neurosci. 25, 3478–3487. 10.1523/JNEUROSCI.3766-04.2005 15800203 PMC6724908

[B23] Heng M. Y. Lin S. T. Verret L. Huang Y. Kamiya S. Padiath Q. S.. (2013). Lamin B1 mediates cell-autonomous neuropathology in a leukodystrophy mouse model. J. Clin. Invest. 123, 2719–2729. 10.1172/jci66737 23676464 PMC3668844

[B24] Hopker V. H. Shewan D. Tessier-Lavigne M. Poo M. Holt C. (1999). Growth-cone attraction to netrin-1 is converted to repulsion by laminin-1. Nature 401, 69–73. 10.1038/43441 10485706

[B25] Ide C. (1996). Peripheral nerve regeneration. Neurosci. Res. 25, 101–121. 10.1016/0168-0102(96)01042-5 8829147

[B26] Iruarrizaga-Lejarreta M. Varela-Rey M. Lozano J. J. Fernández-Ramos D. Rodríguez-Ezpeleta N. Embade N.. (2012). The RNA-binding protein human antigen R controls global changes in gene expression during Schwann cell development. J. Neurosci. 32, 4944–4958. 10.1523/JNEUROSCI.5868-11.2012 22492050 PMC3331722

[B27] Jessen K. R. Mirsky R. (2008). Negative regulation of myelination: relevance for development, injury, and demyelinating disease. Glia 56, 1552–1565. 10.1002/glia.20761 18803323

[B28] Jessen K. R. Mirsky R. (2016). The repair Schwann cell and its function in regenerating nerves. J. Physiol. 594, 3521–3531. 10.1113/JP270874 26864683 PMC4929314

[B29] Jessen K. R. Mirsky R. Lloyd A. C. (2015). Schwann cells: development and role in nerve repair. Cold Spring Harb. Perspect. Biol. 7:a020487. 10.1101/cshperspect.a020487 25957303 PMC4484967

[B30] Kim D. Pertea G. Trapnell C. Pimentel H. Kelley R. Salzberg S. L. (2013). TopHat2: accurate alignment of transcriptomes in the presence of insertions, deletions and gene fusions. Genome Biol. 14:R36. 10.1186/gb-2013-14-4-r36 23618408 PMC4053844

[B31] Kim Y. Remacle A. G. Chernov A. V. Liu H. Shubayev I. Lai C.. (2012). The MMP-9/TIMP-1 axis controls the status of differentiation and function of myelin-forming Schwann cells in nerve regeneration. PLoS One 7:e33664. 10.1371/journal.pone.0033664 22438979 PMC3306282

[B32] Lin C. R. Yang C. H. Huang C. E. Wu C. H. Chen Y. S. Sheen-Chen S. M.. (2011). GADD45A protects against cell death in dorsal root ganglion neurons following peripheral nerve injury. J. Neurosci. Res. 89, 689–699. 10.1002/jnr.22589 21337369

[B33] Liu M. Sun A. Shin E. J. Liu X. Kim S. G. Runyons C. R.. (2006). Expression of microsomal epoxide hydrolase is elevated in Alzheimer’s hippocampus and induced by exogenous β-amyloid and trimethyl-tin. Eur. J. Neurosci. 23, 2027–2034. 10.1111/j.1460-9568.2006.04724.x 16630050

[B34] Liu X. Zhao Y. Peng S. Zhang S. Wang M. Chen Y.. (2016). BMP7 retards peripheral myelination by activating p38 MAPK in Schwann cells. Sci. Rep. 6:31049. 10.1038/srep31049 27491681 PMC4974506

[B35] Love M. I. Huber W. Anders S. (2014). Moderated estimation of fold change and dispersion for RNA-seq data with DESeq2. Genome Biol. 15:550. 10.1186/s13059-014-0550-8 25516281 PMC4302049

[B36] Madl C. M. Heilshorn S. C. (2015). Matrix interactions modulate neurotrophin-mediated neurite outgrowth and pathfinding. Neural Regen. Res. 10, 514–517. 10.4103/1673-5374.155426 26170800 PMC4424732

[B37] Maldonado E. N. Alderson N. L. Monje P. V. Wood P. M. Hama H. (2008). FA2H is responsible for the formation of 2-hydroxy galactolipids in peripheral nervous system myelin. J. Lipid Res. 49, 153–161. 10.1194/jlr.m700400-jlr200 17901466 PMC2662131

[B38] Mei L. Xiong W. C. (2008). Neuregulin 1 in neural development, synaptic plasticity and schizophrenia. Nat. Rev. Neurosci. 9, 437–452. 10.1038/nrn2392 18478032 PMC2682371

[B39] Michailov G. V. Sereda M. W. Brinkmann B. G. Fischer T. M. Haug B. Birchmeier C.. (2004). Axonal neuregulin-1 regulates myelin sheath thickness. Science 304, 700–703. 10.1126/science.1095862 15044753

[B40] Mirsky R. Woodhoo A. Parkinson D. B. Arthur-Farraj P. Bhaskaran A. Jessen K. R. (2008). Novel signals controlling embryonic Schwann cell development, myelination and dedifferentiation. J. Peripher. Nerv. Syst. 13, 122–135. 10.1111/j.1529-8027.2008.00168.x 18601657

[B41] Miyamoto T. Morita K. Takemoto D. Takeuchi K. Kitano Y. Miyakawa T.. (2005). Tight junctions in Schwann cells of peripheral myelinated axons: a lesson from claudin-19-deficient mice. J. Cell Biol. 169, 527–538. 10.1083/jcb.200501154 15883201 PMC2171943

[B42] Morrissey T. K. Kleitman N. Bunge R. P. (1991). Isolation and functional characterization of Schwann cells derived from adult peripheral nerve. J. Neurosci. 11, 2433–2442. 10.1523/JNEUROSCI.11-08-02433.1991 1869923 PMC6575499

[B43] Namgung U. (2014). The role of Schwann cell-axon interaction in peripheral nerve regeneration. Cells Tissues Organs 200, 6–12. 10.1159/000370324 25765065

[B44] Parkinson D. B. Bhaskaran A. Arthur-Farraj P. Noon L. A. Woodhoo A. Lloyd A. C.. (2008). c-Jun is a negative regulator of myelination. J. Cell Biol. 181, 625–637. 10.1083/jcb.200803013 18490512 PMC2386103

[B45] Parkinson D. B. Bhaskaran A. Droggiti A. Dickinson S. D’Antonio M. Mirsky R.. (2004). Krox-20 inhibits Jun-NH2-terminal kinase/c-Jun to control Schwann cell proliferation and death. J. Cell Biol. 164, 385–394. 10.1083/jcb.200307132 14757751 PMC2172235

[B46] Patodia S. Raivich G. (2012). Role of transcription factors in peripheral nerve regeneration. Cell Tissue Res. 5:8. 10.3389/fnmol.2012.00008 22363260 PMC3277281

[B47] Pereira J. A. Lebrun-Julien F. Suter U. (2012). Molecular mechanisms regulating myelination in the peripheral nervous system. Trends Neurosci. 35, 123–134. 10.1016/j.tins.2011.11.006 22192173

[B48] Ronchi G. Haastert-Talini K. Fornasari B. E. Perroteau I. Geuna S. Gambarotta G. (2016). The Neuregulin1/ErbB system is selectively regulated during peripheral nerve degeneration and regeneration. Eur. J. Neurosci. 43, 351–364. 10.1111/ejn.12974 26061116

[B49] Shin Y. K. Jang S. Y. Park J. Y. Park S. Y. Lee H. J. Suh D. J.. (2013). The Neuregulin-Rac-MKK7 pathway regulates antagonistic c-jun/Krox20 expression in Schwann cell dedifferentiation. Glia 61, 892–904. 10.1002/glia.22482 23505039

[B50] Siebert H. Kahle P. J. Kramer M. L. Isik T. Schlüter O. M. Schulz-Schaeffer W. J.. (2010). Over-expression of α-synuclein in the nervous system enhances axonal degeneration after peripheral nerve lesion in a transgenic mouse strain. J. Neurochem. 114, 1007–1018. 10.1111/j.1471-4159.2010.06832.x 20524960

[B51] Sobue G. Shuman S. Pleasure D. (1986). Schwann cell responses to cyclic AMP: proliferation, change in shape and appearance of surface galactocerebroside. Brain Res. 362, 23–32. 10.1016/0006-8993(86)91394-6 3002553

[B52] Sonnenberg-Riethmacher E. Miehe M. Riethmacher D. (2015). Promotion of periostin expression contributes to the migration of Schwann cells. J. Cell Sci. 128, 3345–3355. 10.1242/jcs.174177 26187852

[B53] Stassart R. M. Fledrich R. Velanac V. Brinkmann B. G. Schwab M. H. Meijer D.. (2013). A role for Schwann cell-derived neuregulin-1 in remyelination. Nat. Neurosci. 16, 48–54. 10.1038/nn.3281 23222914

[B54] Syed N. Reddy K. Yang D. P. Taveggia C. Salzer J. L. Maurel P.. (2010). Soluble neuregulin-1 has bifunctional, concentration-dependent effects on Schwann cell myelination. J. Neurosci. 30, 6122–6131. 10.1523/JNEUROSCI.1681-09.2010 20427670 PMC2870719

[B55] Taveggia C. (2016). Schwann cells-axon interaction in myelination. Curr. Opin. Neurobiol. 39, 24–29. 10.1016/j.conb.2016.03.006 27089429

[B56] Taveggia C. Zanazzi G. Petrylak A. Yano H. Rosenbluth J. Einheber S.. (2005). Neuregulin-1 type III determines the ensheathment fate of axons. Neuron 47, 681–694. 10.1016/j.neuron.2005.08.017 16129398 PMC2387056

[B57] Václavíková R. Hughes D. J. Souček P. (2015). Microsomal epoxide hydrolase 1 (EPHX1): gene, structure, function, and role in human disease. Gene 571, 1–8. 10.1016/j.gene.2015.07.071 26216302 PMC4544754

[B58] Vandesompele J. De Preter K. Pattyn F. Poppe B. Van Roy N. De Paepe A.. (2002). Accurate normalization of real-time quantitative RT-PCR data by geometric averaging of multiple internal control genes. Genome Biol. 3:RESEARCH0034. 10.1186/gb-2002-3-7-research0034 12184808 PMC126239

[B59] Wakatsuki S. Araki T. Sehara-Fujisawa A. (2014). Neuregulin-1/glial growth factor stimulates Schwann cell migration by inducing α5 β1 integrin-ErbB2-focal adhesion kinase complex formation. Genes Cells 19, 66–77. 10.1111/gtc.12108 24256316

[B60] Warner L. E. Mancias P. Butler I. J. McDonald C. M. Keppen L. Koob K. G.. (1998). Mutations in the early growth response 2 (EGR2) gene are associated with hereditary myelinopathies. Nat Genet 18, 382–384. 10.1038/ng0498-382 9537424

[B61] Woodhoo A. Alonso M. B. Droggiti A. Turmaine M. D’Antonio M. Parkinson D. B.. (2009). Notch controls embryonic Schwann cell differentiation, postnatal myelination and adult plasticity. Nat. Neurosci. 12, 839–847. 10.1038/nn.2323 19525946 PMC2782951

[B62] Xiang H. Yuan L. Gao X. Alexander P. B. Lopez O. Lau C.. (2017). UHRF1 is required for basal stem cell proliferation in response to airway injury. Cell Discov. 3:17019. 10.1038/celldisc.2017.19 28626588 PMC5468773

[B63] Yi S. Tang X. Yu J. Liu J. Ding F. Gu X. (2017). Microarray and qPCR analyses of wallerian degeneration in rat sciatic nerves. Front. Cell. Neurosci. 11:22. 10.3389/fncel.2017.00022 28239339 PMC5301003

[B65] Yu J. Gu X. Yi S. (2016). Ingenuity pathway analysis of gene expression profiles in distal nerve stump following nerve injury: insights into wallerian degeneration. Front. Cell. Neurosci. 10:274. 10.3389/fncel.2016.00274 27999531 PMC5138191

[B64] Yu G. Wang L. G. Han Y. He Q. Y. (2012). clusterProfiler: an R package for comparing biological themes among gene clusters. OMICS 16, 284–287. 10.1089/omi.2011.0118 22455463 PMC3339379

[B66] Zanazzi G. Einheber S. Westreich R. Hannocks M. J. Bedell-Hogan D. Marchionni M. A.. (2001). Glial growth factor/neuregulin inhibits Schwann cell myelination and induces demyelination. J. Cell Biol. 152, 1289–1299. 10.1083/jcb.152.6.1289 11257128 PMC2199210

